# Laser Scan Compression for Rail Inspection

**DOI:** 10.3390/s24206722

**Published:** 2024-10-19

**Authors:** Jeremiasz Hauck, Piotr Gniado

**Affiliations:** Nevomo IoT, 03-828 Warsaw, Poland; p.gniado@nevomo.tech

**Keywords:** data compression, triangulation laser scanners, railway, rail geometry

## Abstract

The automation of rail track inspection addresses key issues in railway transportation, notably reducing maintenance costs and improving safety. However, it presents numerous technical challenges, including sensor selection, calibration, data acquisition, defect detection, and storage. This paper introduces a compression method tailored for laser triangulation scanners, which are crucial for scanning the entire rail track, including the rails, rail fasteners, sleepers, and ballast, and capturing rail profiles for geometry measurement. The compression technique capitalizes on the regularity of rail track data and the sensors’ limited measurement range and resolution. By transforming scans, they can be stored using widely available image compression formats, such as PNG. This method achieved a compression ratio of 7.5 for rail scans used in the rail geometry computation and maintained rail gauge reproducibility. For the scans employed in defect detection, a compression ratio of 5.6 was attained without visibly compromising the scan quality. Lossless compression resulted in compression ratios of 5.1 for the rail geometry computation scans and 3.8 for the rail track inspection scans.

## 1. Introduction

Rail tracks, crucial transportation infrastructure components, require consistent maintenance to ensure safety and reliability. Defects in rail tracks represent a significant risk factor for train accidents [1]. Traditionally, human inspectors or track-recording vehicles, requiring human operators, conduct inspections [2]. However, these inspections necessitate the temporary closure of rail tracks, disrupting operations and requiring careful scheduling [3].

Integrating inspection devices onto conventional passenger or freight trains offers a potential solution, eliminating the need for additional labor and minimizing track closures. Moreover, employing advanced algorithms enhances the detection accuracy and speed, reducing the risk of human error [4]. Another application of measurement devices attached to trains is in the creation of a digital twin of the rail infrastructure, which is valuable for asset management [5].

Furthermore, demographic trends, such as declining birth rates and an aging workforce, highlight the urgency of automating rail track inspection for the sustainable future of railway transportation [6].

Many rail track inspection methods rely on various sensor technologies [7]. One prevalent approach involves analyzing captured point clouds of rail tracks or processing point clouds for rail geometry measurement. Laser optical sensors are used to acquire point cloud data. Apart from being the input for defect detection algorithms, these data serve multiple purposes, including algorithm development, model training, and human confirmation of defect detection. However, efficient data storage necessitates compression due to the substantial volume of data generated. Unlike image data, a standard compression method for point clouds is relatively nascent, with a recent publication [8]. Despite its promise, stable libraries implementing this standard are scarce, with only a test model provided by the Moving Picture Experts Group currently available [9].

This paper proposes a straightforward compression method for rail track point clouds and rail profile point clouds, leveraging the widely used PNG image compression format. The proposed approach capitalizes on the predictable nature of rail track data and the limited range and precision of laser triangulation sensors to achieve enhanced compression levels. The method encodes each frame of rail track scans into two PNG images: one image represents the x-component and the other represents the z-component of the scan point coordinates. Each scan point coordinate corresponds to a pixel in the image. To convert the floating-point representation of the scan point coordinates into pixel values, the coordinates are first scaled to shift the fractional part into the integer range. The values are then truncated to integers and split into three bytes to fit within the RGB channels of a PNG image. In order to recover the scan, inverse operations are applied to the two images. This conversion can be lossless due to the limited range and resolution of laser triangulation scanners.

While converting point clouds to images is not novel, this study marks the first application of such an approach to laser triangulation scanner measurements and rail track data. Previous works, such as [10], explored converting LiDAR data into an image format, subsequently compressing it using common image compression algorithms. This method utilizes equirectangular projection to map 3D data points onto an image plane, encoding the distance traveled by the LiDAR laser ray until it intersects with an object as a 24-bit number distributed across the red, green, and blue channels.

Similarly, in [11], a technique was introduced to store Terrestrial Laser Scanning (TLS) data in an image format, followed by compression using JPEG-2000. This method utilizes raw sensor data, enabling direct conversion to an image format due to the regularity of the sampling pattern. Any calibration offsets lost due to assuming a perfect grid sampling pattern are preserved as image metadata. Although JPEG-2000 supports the compression of images with floating-point pixel values, the author opted for integer values for pixel representation.

Another relevant work, [12], proposes a method for compressing 3D LiDAR scan data from autonomous vehicles. This technique, akin to [11], employs range images to store sequences of 3D scan data. However, it diverges by employing a neural network to predict pixel values based on prior LiDAR scans and neighboring LiDAR measurements already converted to pixel values. The disparity between the predicted and actual pixel values is entropy encoded, yielding lossless compression.

Furthermore, [13] explored the utilization of H.264 for compressing 3D scan range data. The range data for each frame are divided into multiple frames, and these streams are individually compressed using the H.264 standard. Leveraging spatio-temporal relationships in the data, the H.264 compression standard efficiently compresses frame differences, resulting in high compression rates for similar frames.

This paper presents two types of laser scans used in rail track inspection: one from the rail track inspection module and the other from the rail geometry measuring module. The placement and orientation of the laser triangulation scanners within each module, the data acquisition process, and the visualization of the collected scans are discussed for both modules. The proposed laser triangulation scan compression algorithm is then introduced and it was evaluated across various parameters for scans from both the rail track inspection module and the rail geometry measuring module. The analysis showed that the proposed method delivered satisfactory scan quality at compression ratios of 5.6 for the rail track inspection module and 7.5 for the rail geometry measuring module. For lossless compression, the method achieved compression ratios of 3.8 for rail track inspection scans and 5.1 for rail geometry scans.

## 2. Materials and Methods

### 2.1. Laser Scan Measurement and Frame Collection

#### 2.1.1. Laser Triangulation Scanner

Various methods were devised for non-contact range measurements [14]. With the advancement of autonomous driving technology, LiDAR sensors have garnered significant attention in recent years [15]. Off-the-shelf LiDAR-based scanning systems tailored for rail infrastructure measurement have been developed [16]. LiDAR measurements, as described in [17], are primarily utilized for medium- to long-distance range measurements. One example of using these types of sensors is the creation of a digital twin of the railway infrastructure, as described in [5,18]. However, for rail track scanning, where sensors can be mounted beneath the train near the track, triangulation-based sensors offer exceptional precision for short-distance range measurements [17]. Many commercially available solutions for track inspection, such as those presented in [19,20], rely on laser triangulation scanners.

These sensors were proven to be accurate enough to detect squat defects on rail surfaces, including the localization and size determination of defects that measure only a few square millimeters [21]. Another engineering study [22] demonstrated the potential of detecting sub-millimeter rail surface defects using laser scanners. Several studies also highlighted the possibility of integrating these sensors with machine learning models to inspect various components of the railway, such as detecting the presence and condition of rail fasteners [23], marking the running surface of the rail profile [24], or identifying changes on the railway between consecutive scans, including sleeper skew, incorrect ballast volume, and the addition or removal of fasteners and anchors [25]. The application of laser-based sensors for rail geometry measurement has been discussed in [26,27,28].

The operating principle of triangulation sensors, as explained in [17], highlights their suitability for rail inspection. Triangulation sensors offer high measurement precision at short ranges and have no moving parts, which reduces potential issues in vibration-prone environments. Additionally, these sensors capture all scan points simultaneously by using CMOS imaging sensors with a global shutter, ensuring perfectly aligned cross-section scans of the entire rail track, regardless of the train’s speed. Moreover, the CMOS imaging sensors in these scanners, with their high sample rates, allow for dense scan data collection. The scan density can be maintained if the product of the desired cross-section scan count per meter and the train’s speed (in meters per second) is less than the sensor’s sample rate.

Adjusting the optical system parameters and the image sensor’s position relative to the laser beam allows for different measurement ranges and accuracies. Integrated sensors with imaging sensors and a laser source are available on the market [29,30,31], along with single cameras dedicated to triangulation scan measurement for customized scanner configurations that require an external laser source [32]. However, configuring a custom laser triangulation scanner necessitates calibration [33].

#### 2.1.2. Rail Track Inspection Scans

Rail infrastructure comprises various components, including rail tracks, signaling systems, and high-voltage lines, each requiring specific types of measuring devices. This paper focuses solely on rail track infrastructure, encompassing the rails, rail fasteners, sleepers, and railway ballast.

**Figure 1:**
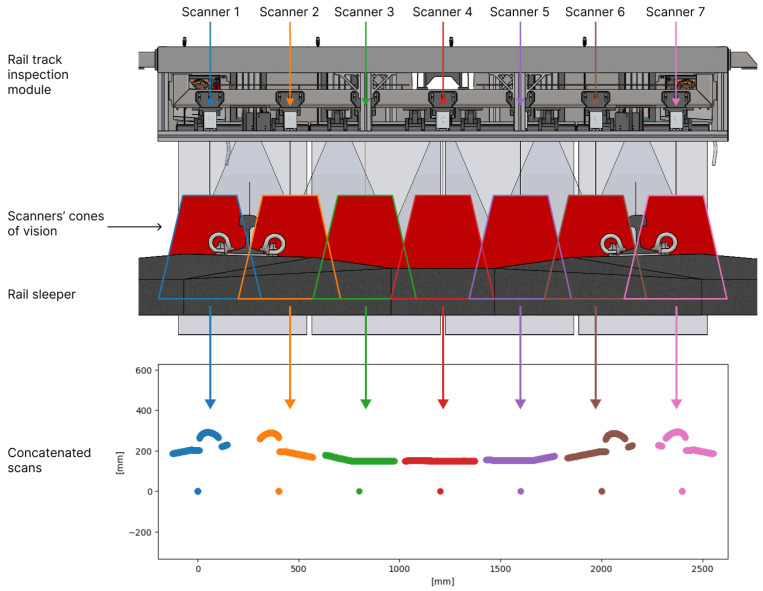
Rail track inspection module with the triangulation sensors’ overlapping cones of vision and a cross-section of the scanned rail track at the top, and the captured cross-section scan by the rail track inspection module at the bottom.

Off-the-shelf laser scanners were selected for the developed rail track infrastructure measurement device. Figure 1 shows the resulting sensor configuration, with each sensor’s measurement range represented by the colored trapezoids. The overlapping measurement ranges ensure that taller objects stay within range, preventing them from falling out of view at the edges of each sensor’s field of vision. Triangulation scanners have a tapered cone of vision, with the laser beam intersecting the camera’s field of view at both the top and bottom as it enters and exits this cone. A total of seven scanners were used in the rail track inspection module. Since this module was not designed for rail head inspection, no scanners were positioned to capture that part of the rail track.

A single measurement from the rail track inspection module consists of concatenated scans from each sensor. Due to the overlapping fields of view of the scanners, certain parts of the scan are captured redundantly. The plot at the bottom of Figure 1 shows a scatter plot of the point clouds from all sensors, transformed into a shared reference frame, with each sensor’s point cloud depicted in a different color. The scans from different sensors are laterally offset based on their measurement ranges. The first sensor, labeled “Scanner 1”, captured the blue section of the concatenated scan. By comparing the cross-section drawing of the rail sleeper with the bottom plot, one can observe the resemblance between the rail sleeper and fasteners and the captured scan. The specific laser scanner model used assigns the coordinates of the sensor’s reference frame origin—zero on both the lateral and vertical axes—to any points that exceed the measurement range. As a result, beneath each scan in the bottom plot of Figure 1, there is a cluster of points at zero height that does not correspond to any physical object.

#### 2.1.3. Rail Geometry Scans

**Figure 2:**
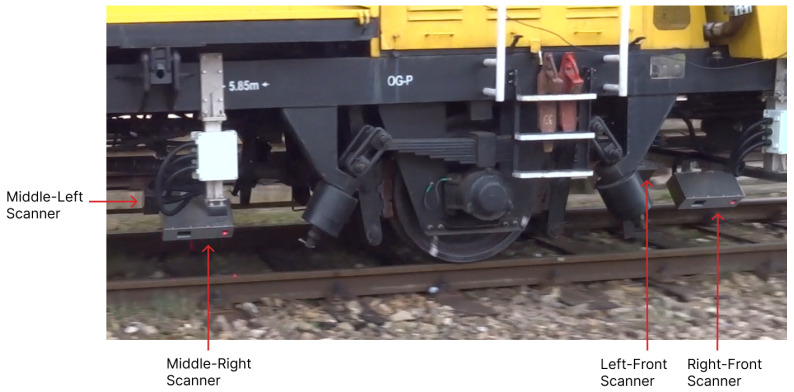
Four of the six rail-geometry-measuring module scanners attached to a rail maintenance vehicle.

One method for measuring rail geometry is the chord method, also known as the versine method [34,35]. This method uses three longitudinally spaced points on the rail to determine its curvature. In non-contact chord measurements, laser scanners capture these three points. Six laser scanners are required to measure the geometry of both rails, with three scanners dedicated to each rail. Figure 2 shows a general-purpose rail maintenance vehicle equipped with rail-geometry-measuring module scanners. Only four of the six scanners are visible in the figure; the other two are located at the rear of the vehicle. The scanners are labeled based on their attachment positions relative to the vehicle and each other. They are angled to view one side of the rail.

**Figure 3:**
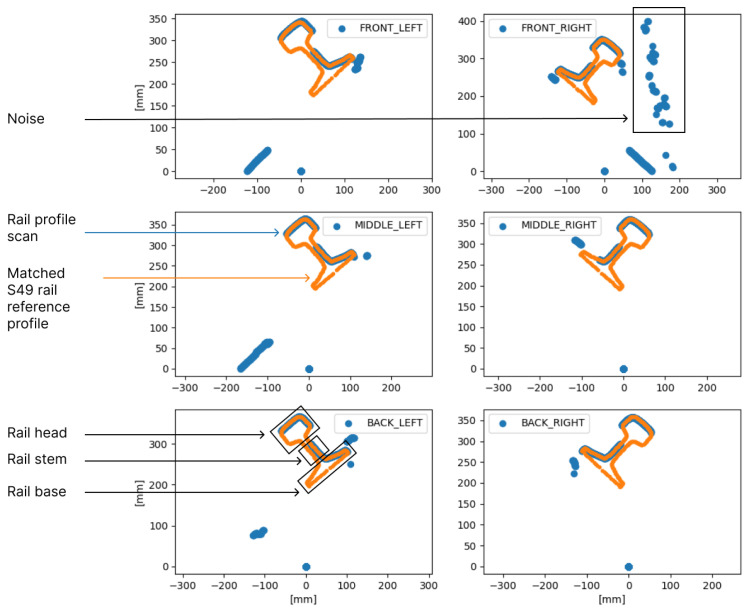
One rail scan sample by the rail-geometry-measuring module. The raw scan points are shown in blue, and the matched S49 rail profile points are in orange.

Figure 3 shows a scatter plot of a sample of rail profiles collected by the rail-geometry-measuring module. These profiles exhibited a tilt because the scanners captured the rail at an angle, where they measured both the rolling surface of the rail head and its side surface. Matched S49 rail reference profiles are plotted in orange over the collected scans, which are shown in blue. Automatically matching a reference profile to the measured scan is a crucial step in rail geometry measurement, as it determines the rail’s position relative to the scanner. In this case, the matched reference profiles were plotted to illustrate which parts of the rail were captured by the rail geometry scanners. Only one side of the rail was captured due to the scanner’s orientation; nevertheless, this was sufficient for accurately matching the reference profile to the measured scan. The measurements in Figure 3 are shown in the scanner’s frame of reference. The top-right scan in Figure 3 contains a significant amount of noise, which is expected in rail profile scans due to various measurement conditions, such as polished reflective surfaces, unpolished dark steel, intense sunlight, or a lack of sunlight.

#### 2.1.4. Scan Fusion

Although scan fusion falls outside the scope of this paper, it is important to note that it is not a particularly challenging task. A straightforward approach involves aligning each sensor’s measurements to a shared reference frame and laterally concatenating the transformed scans. The concatenated data can then be sorted based on the lateral coordinate. Since the concatenated scans are partially sorted, sorting is only necessary for samples that may overlap with data from another sensor. The sorted concatenated scan can subsequently be resampled to maintain a consistent point density throughout the scan, and resampling also facilitates the interpolation of any missing samples.

However, it is important to highlight that no scan fusion was applied in the development stage of the measuring device.

#### 2.1.5. Frame Collection

A single measurement from a scanner yields a two-dimensional array representing a lateral slice of the rail track. To construct a 3D point cloud, multiple single measurements are aggregated to form a frame, resulting in a three-dimensional array. The spacing between consecutive slices in the frame is determined by the triggering signal frequency, ideally increasing proportionally with the train speed. This ensures that the slices maintain a constant separation, regardless of the train’s speed, as long as the product of the desired slice count per meter and the train’s speed (in meters per second) is less than the sensor’s sample rate. Since the spacing between the slices in the longitudinal direction remains constant, it is unnecessary to store this value for every scan point; instead, a single value per file in the metadata suffices. However, it is essential to note that such a frame is neither a true point cloud, as the 3D points have only two coordinates, nor a true depth map, as each point possesses a second coordinate apart from the distance value.

**Figure 4:**
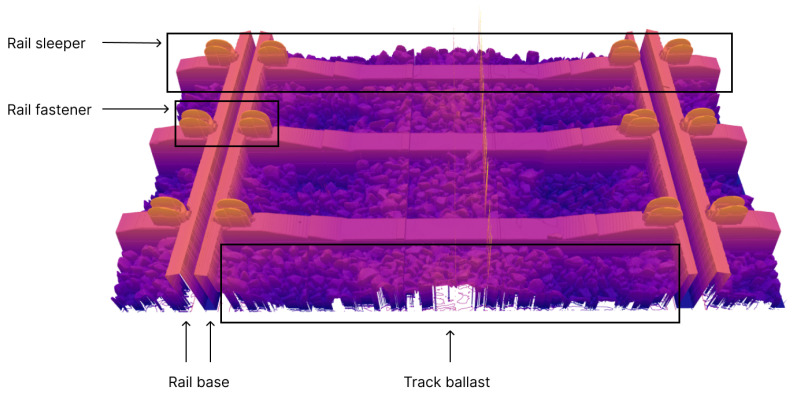
Rail track inspection scan frame 3D render.

**Figure 5:**
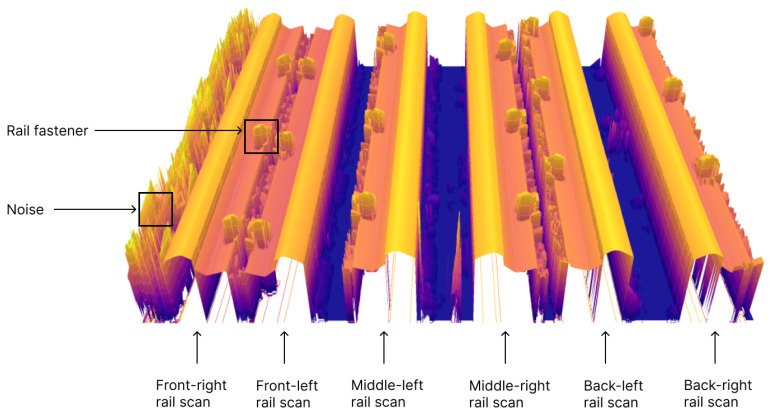
Rail scan for rail geometry measurement frame 3D render.

Figure 4 presents a 3D visualization of a collected frame from the rail track inspection module. The 3D render shows the track ballast, rail sleepers, rail fasteners, and the rail base of the two rails. Since the rail heads of both rails were intentionally placed outside the measurement range of any scanner, they are not visible in Figure 4. Additionally, due to the overlapping fields of view of the scanners, as explained in Section 2.1.2, the rail sleepers appear uneven at the concatenation points of scans captured by different sensors. Meanwhile, Figure 5 depicts a 3D render of a collected frame from the rail-geometry-measuring module. This frame included six columns of rail scans: three columns for the left rail, each representing different longitudinal offsets, and three columns for the right rail at corresponding offsets. Each column corresponds to one scanner in the rail geometry measuring module. Because the scanners were oriented at an angle to the rail, only one side of the rail profile was captured, which resulted in tilted scans. Notably, the rail profile scans from the rail geometry measuring module exhibited greater uniformity compared with the scans of the fasteners, sleepers, and rail ballast.

### 2.2. Compression Algorithm

#### 2.2.1. General Description of the Compression Algorithm

The primary concept of the algorithm involves leveraging conventional image compression techniques to compress three-dimensional point clouds. A secondary idea for enhancing the compression efficiency is to exploit the spatial uniformity inherent in rail track scans through delta encoding. Additionally, the algorithm utilizes number scaling to facilitate the conversion to an image format and to filter out noise from the measurements.

**Figure 6:**
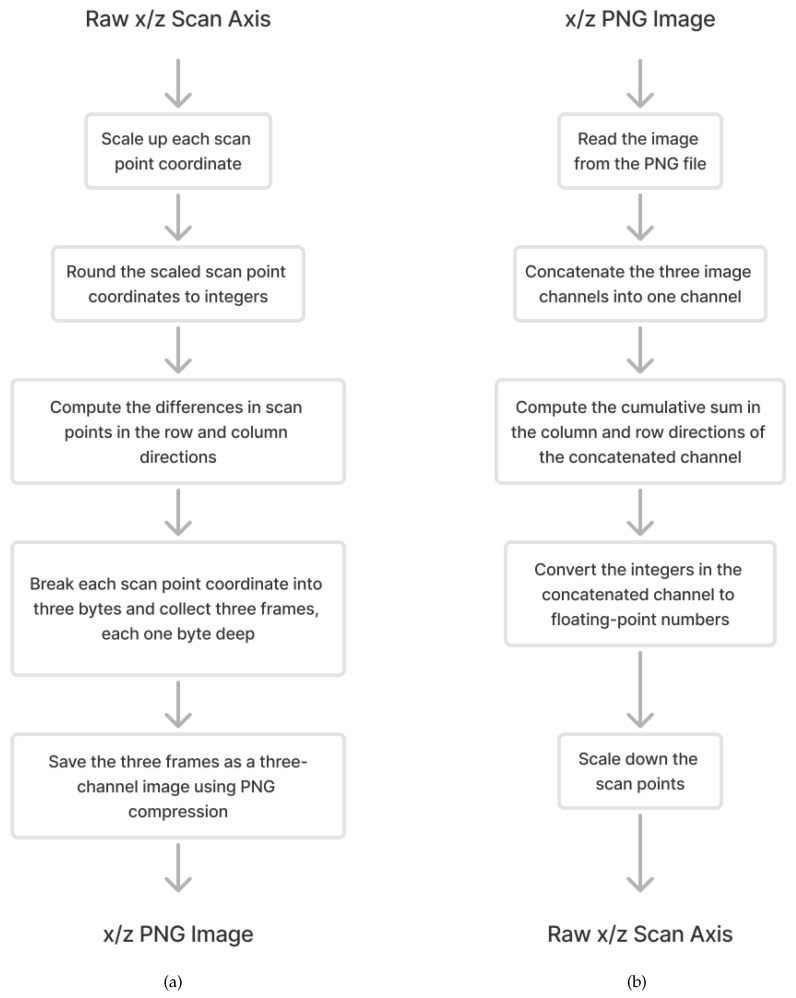
Scan compression and decompression method block diagrams; (**a**) Scan-to-image compression method block diagram; (**b**) Image-to-scan decompression method block diagram.

Figure 6a depicts a block diagram of the proposed compression method for rail track laser scans. Each step in the block diagram is further elaborated in the following paragraphs.

#### 2.2.2. Range Conversion

The laser scanners utilized in rail track monitoring employ triangulation to gauge the distance to objects. These measurements are typically expressed as floating-point numbers, given the continuous nature of the measured distance. However, most image compression formats anticipate integer values for pixels, necessitating a conversion from one numerical format to another for storage.

To convert floating-point numbers to integers, these numbers must be multiplied by a scaling factor and then truncated to an integer format. Subsequently, the same scaling factor must be applied to divide the integer number to revert it to the original float value. While this operation does result in information loss unless the scaling factor is sufficiently large, the finite resolution of sensors ensures that a relatively modest scaling factor can retain all the information in the measurements, rendering the conversion lossless from the perspective of sensor resolution but lossy concerning the original float value.

Importantly, as the minimum and maximum distances bind the measuring range of laser triangulation scanners, there is no risk of overflow when multiplied by the scaling factor. Furthermore, the numerical scaling factor can be intentionally set to a lower value if a reduced resolution suffices while a higher compression ratio is desired.

#### 2.2.3. Delta Encoding

Given that the scanners are mounted on vehicles moving along rail tracks, the relative motion between the scanners and the rail track in the lateral direction is minimal. As a result, consecutive scans demonstrate a high degree of similarity, creating an opportunity for enhanced compression efficiency.

The proposed method leverages this similarity by storing the differences in scans in two directions of the collected frame: longitudinal and lateral. This process, known as delta encoding [36], computes the differences between consecutive measurements, resulting in values with a smaller range than the original frame. As a result, subsequent image compression becomes more effective.

Although PNG compression incorporates its delta encoding filters, some of which perform similar operations to the one described, no filtering is applied to three-channel images [37]. Therefore, delta encoding is an additional step in the presented method, enhancing the compression efficiency.

#### 2.2.4. Conversion to Image Format

The laser triangulation scanners produce a fixed-length array of measurements, with each element comprising depth and lateral position components. The consistent number of points in the measurement array ensures uniform-sized collected frames. Treating each scan point as a pixel facilitates the conversion of measured frames into images.

When the measurements are converted to an integer format, they occupy more bits than expected for an image compression procedure, typically 8 bits per channel. However, images can accommodate three or even four channels, including an alpha channel, providing 24 or 32 usable bits per image pixel. To store the measured value expressed as an integer, it is divided into bytes, with each byte occupying a different channel of the created image. The most significant byte is discarded if only three channels are used. This process prepares the image for compression.

The PNG standard is employed to achieve lossless data compression. The same compression procedure is applied to both coordinates for each measurement, resulting in two images per collected frame of the scans.

### 2.3. Decompression

The decompression procedure, depicted in Figure 6b, reverses all the steps outlined in Figure 6a, executing them in the opposite order.

## 3. Results

The compression algorithm was tested on data gathered from two inspection device modules: the rail track inspection module and the rail geometry measuring module. The former collected scans of the entire rail track, including the rails fasteners, sleepers, and ballast, using seven scanners, while the latter gathered one-sided profiles of both rails at three points simultaneously with six scanners.

### 3.1. Rail Track Inspection Scan Compression Tests

The compression algorithm underwent testing on raw data captured by the prototyped rail track inspection module. The data originated from a railway siding used for parking and servicing trains that featured a variety of elements, such as concrete and wooden sleepers, different rail fasteners, straight rail sections, and railway switches.

The following compression parameters were varied to assess their impact on the compression rate of the algorithm:

- Precision scaler;
- Frame length;
- Compression approach: compressing scans from each sensor independently vs. compressing the concatenated scans from all sensors.

The compression algorithm was evaluated in two ways: visually assessing how the compression affected the scan and quantifying the difference between the compressed and raw scan values. For the evaluation, 200,000 single-line scans were compressed using different values of the numerical precision scaler. The frames comprised 200 single scan lines, with each scanner’s measurements compressed and saved independently.

**Figure 7:**
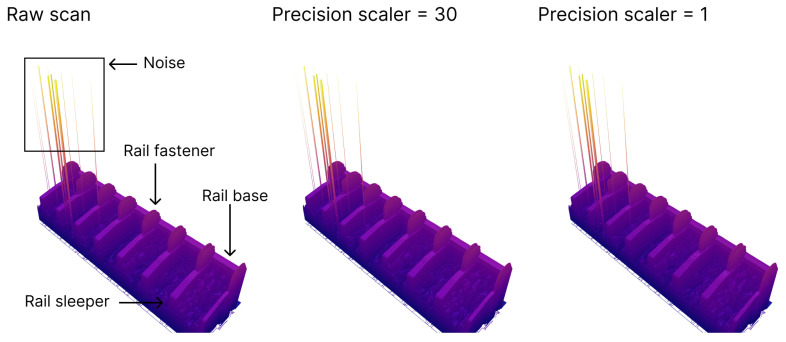
Comparison of the rail track inspection scans from one sensor with different levels of compression applied to the scans.

**Figure 8:**
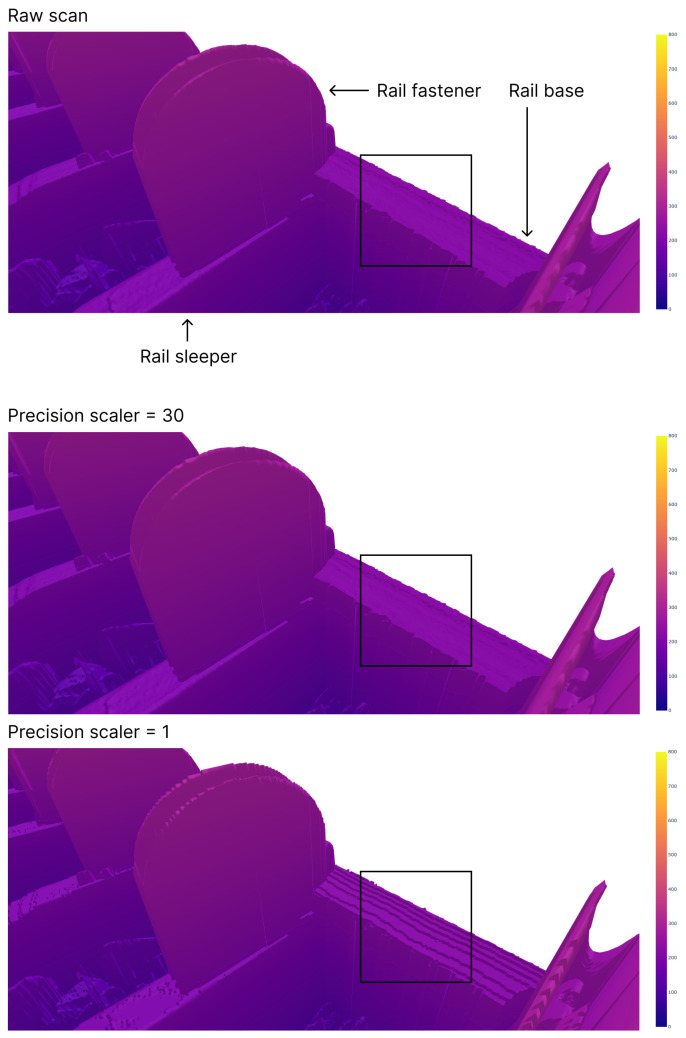
Closeup of the rail track inspection scan fragments from one sensor with different levels of compression applied to the scans.

Figure 7 illustrates the same scan frame collected by one of the sensors, presented in both its raw form and in a compressed format using various numerical precision scalers. At this distance, no discernible differences were evident between the scans. Importantly, the noise in the scan was retained, and no artifacts were introduced during the compression process. However, a closer examination in Figure 8 revealed that a precision scaler of one significantly degraded the scan to the point where differences became noticeable. This was particularly evident when comparing the area of the scans enclosed by the black box in Figure 8. A precision scaler of one effectively truncated measurements below one millimeter to zero. Consequently, the steps visible in the scan region bounded by the black box for the precision multiplier of one had heights that were integer values in millimeters. In contrast, with a precision scaler value of 30, the compressed scan remained indistinguishable from the raw scan, even upon closer inspection. A scaler of 30 preserved part of the measurement’s fractional value between 0.1 and 0.01 mm, meaning a much greater magnification was required to discern any differences between the raw scan and the compressed scan.

**Figure 9:**
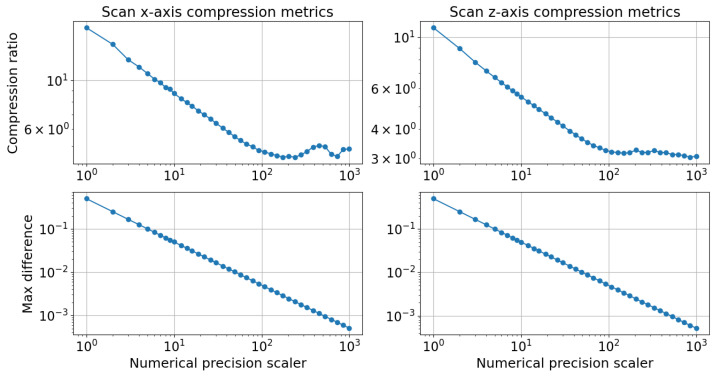
Rail track inspection scan compression metrics, which consisted of the compression ratio and the maximum absolute difference between the raw scan and the compressed scan samples for both axes.

A sweep test was conducted to analyze the effect of varying the numerical precision scaler from 1 to 1000. The results are presented in Figure 9, with each column of plots corresponding to one axis, analyzed independently.

The first row of plots illustrates the compression level as a function of the numerical precision scaler. The compression level is computed as the ratio of the sum of the raw scan sizes to the sum of the compressed scan sizes. It was observed that the compression level decreased as the numerical precision scaler increased. For instance, with a numerical precision scaler of one, the z-axis was compressed by approximately 11.0 times. In contrast, with a scaler of 1000, the z-axis size was reduced by a factor of 3.1 compared with the original scan size. Considering that the x-axis was more compressible than the z-axis, the combined compression level of both axes, when using the same numerical precision scalers for both axes, was 13.5 for a numerical precision scaler of 1 and 3.8 for a scaler of 1000. Notably, bigger numerical precision scalers did not consistently increase the compression level. Depending on the distribution of the scan values, certain numerical precision scalers may scale fractions into more unique integer parts than others, resulting in a lower compression level.

Furthermore, the compression algorithm was analyzed in terms of the absolute difference between the raw scan sample values and the compressed scan sample values. For each scan, the maximum difference was recorded for a given numerical precision scaler. These results are plotted in Figure 9, indicating that the maximum difference decreased as the values of the numerical precision scaler increased. The relationship between the maximum absolute difference and the numerical precision scaler followed a power function: $\max\;\mathrm{difference}=0.498*{\left(\mathrm{numerical}\;\mathrm{precision}\;\mathrm{scaler}\right)}^{-0.998}$. This relationship was determined using linear regression. The smallest preserved fraction after scaling and rounding was the reciprocal of the numerical precision scaler; for example, for the numerical precision scalers of 10 and 4, the smallest preserved fractions were 0.1 and 0.25, respectively. The maximum possible rounding error when rounding to the nearest integer was 0.5. Consequently, the maximum difference between the raw sample and the rounded scaled sample was half the reciprocal of the numerical precision scaler. The compression ratio as a function of the numerical precision scaler exhibited a power function characteristic for scaler values that ranged from 4 to 70. For values that exceeded 100, the compression tapered off due to the limited resolution of the sensors. Once the numerical precision scaler reached a sufficient level, the entire measurement was preserved, and further increases in the scaler only rescaled the same set of values without adding new values, thereby keeping the compression ratio constant.

**Figure 10:**
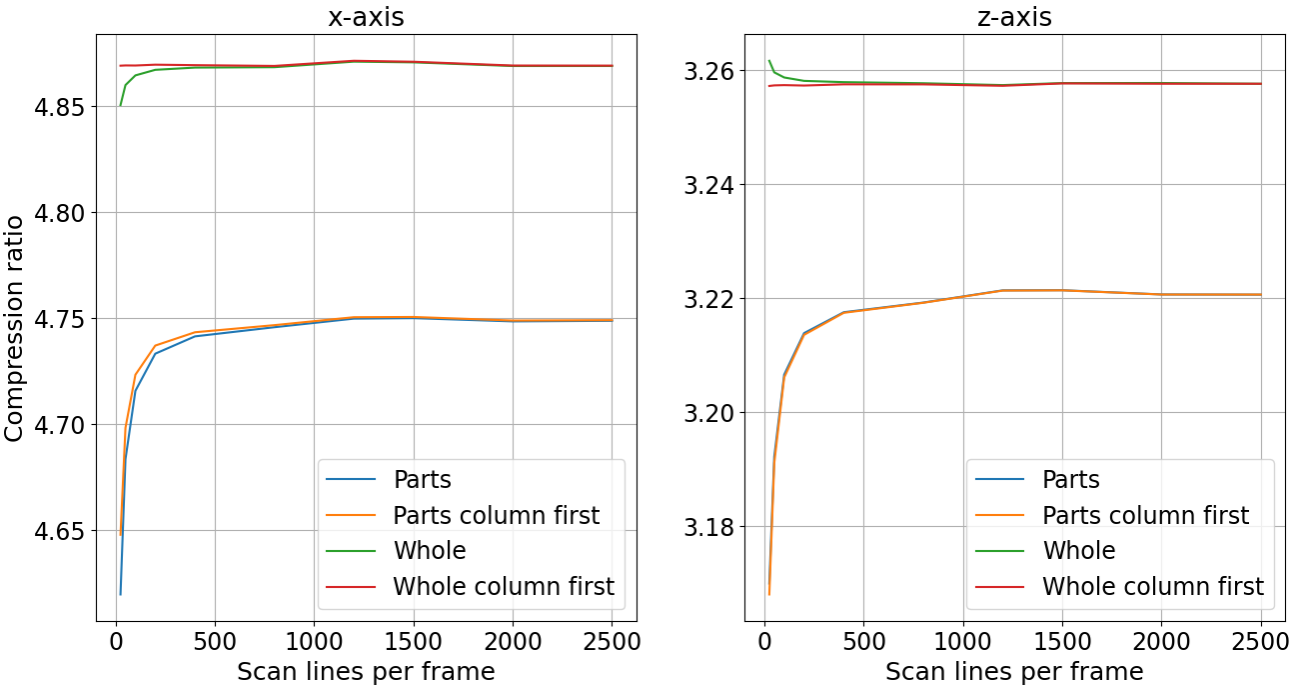
Rail track inspection scan compression level change with frame size and concatenation of sensor measurements.

In Figure 10, the compression level of the rail track inspection scans is plotted as a function of frame size for different compression policies, with the numerical precision scaler set to 100. Scan lines from sensors can be saved independently for each sensor or concatenated into a single scan line. The results for scans saved independently are labeled “Parts” on the plot, whereas the results for concatenated scans are labeled “Whole”. Additionally, the delta encoding difference can be computed first in the row and then in the column direction or vice versa. The results where the difference was first computed in the column direction are labeled “column first” in Figure 10.

From the plots in Figure 10, it can be inferred that concatenating scan lines in the lateral direction from each sensor yielded a better compression ratio compared with saving each sensor’s scan lines independently. For the x-axis, when all the scans were concatenated and the delta encoding difference was first computed in the columns, the compression ratio was approximately 4.87 for all frame sizes. In contrast, when the scans from each sensor were compressed and saved independently, the compression ratios were 4.69 for a frame size of 50 lines and 4.75 for a frame size that exceeded 1000 lines. For the z-axis, concatenated scans achieved a compression ratio of 3.25 across all frame sizes, while independent scans yielded ratios of 3.20 for a frame size of 100 lines and 3.22 for a frame size above 1000 lines. The compression ratio of concatenated scans was less affected by the number of scan lines per frame, providing flexibility in selecting the scan line count based on other requirements.

**Figure 11:**
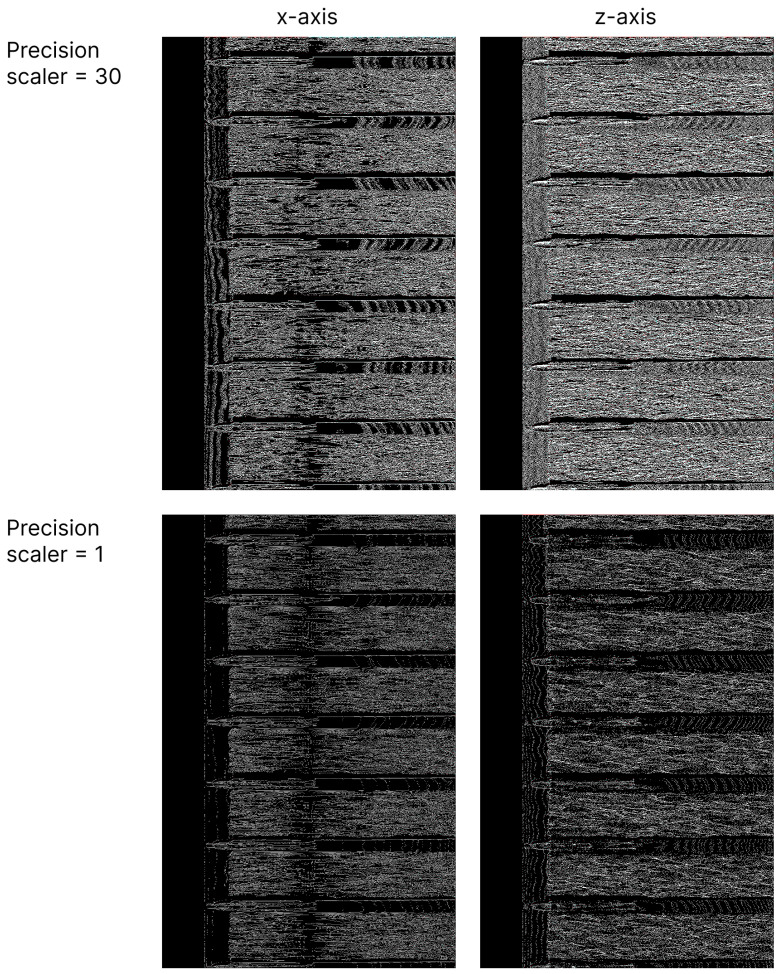
Compressed rail track inspection scans from one sensor into PNG images with different compression levels. Rows of images correspond to different compression levels; columns correspond to different scan axes.

In Figure 11, compressed output PNG images of the same scan fragment as shown in Figure 7 and Figure 8, with precision multipliers of 30 and 1, respectively, are depicted. Before converting the scans into images, the difference was computed in both the row and column directions to minimize the range of possible values.

In the resulting compressed images displayed in Figure 11, the difference values, except for the first sample values, were relatively small compared with the original values, particularly if the original scan values changed smoothly. This characteristic was reflected in the appearance of the compressed images: zero difference values corresponded to pure black pixels, while small positive values typically resulted in black pixels due to only the least significant bits being nonzero. Conversely, small negative values, represented in two’s complement format, had all but the least significant bits set to one, which appeared as white pixels.

Notably, images of scans compressed with a numerical precision scaler of 1 contained significantly more black pixels compared with those compressed with a precision scaler of 30. This occurred because as the numerical precision was reduced, changes between values became less frequent, which led to a higher concentration of pure black pixels in the compressed images. Interestingly, the structure of the rail track could still be deduced from these compressed images; for instance, the horizontal stripes corresponded to rail sleepers. This suggests that the compression ratio could potentially be improved further if this preserved structural information were somehow integrated into the compression algorithm. On the other hand, the ability to discern the rail track structure in the PNG images proved useful when browsing through the files, as it allowed for a quick visual inspection without the need for specialized scan-viewing software.

### 3.2. Rail Geometry Scan Compression Tests

Rail scan data for rail geometry computation were obtained from the same railway siding as the rail track inspection scan data by utilizing a prototyped rail geometry measuring module with scanners positioned differently than those in the rail track inspection module.

Similar to the rail track inspection scans, the compression algorithm underwent testing across various parameters:

- Numerical precision scaler;
- Frame length;
- Compressing scans from every sensor independently vs. compressing concatenated scans from every sensor.

Two hundred thousand single-line scans were compressed for each setting using different numerical precision scaler values.

**Figure 12:**
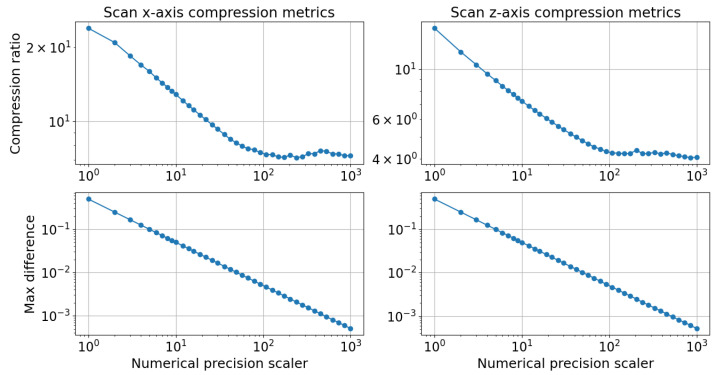
Rail geometry scan compression metrics, which consisted of the compression ratio and the maximum absolute difference between the raw scan and compressed scan samples for both axes.

Figure 12 illustrates the change in the compression ratio with numerical precision scaler values. The absolute difference between the compressed and raw scan points was computed for every frame collected from the scan lines. The worst maximum values were then plotted against the numerical precision scaler.

It was observed that the maximum absolute difference values decreased with increased values of the numerical precision scaler. The plots in Figure 12 are similar to those in Figure 9 presented in Section 3.1. The same arguments presented in Section 3.1 apply to both figures, so they are not repeated here. The rail geometry scan compression was more effective than the rail track inspection scan compression, which could be seen from the comparison of the compression ratio plots in Figure 9 and Figure 12. For the rail geometry scans, the compression ratio was 6.84 for a numerical precision scaler of 30, whereas for the infrastructure scans, it was 5.02 for the same value of numerical precision scaler. A tradeoff between the compression ratio and loss of information could be made by comparing the compression ratio and max difference plots in Figure 12. For instance, decreasing the precision scaler from 100 to 10 increased the compression ratio of the scan z-axis from around 5.4 to around 9.3, while the maximum difference value rose from 0.0045 to 0.05 mm. Notably, the maximum difference increased tenfold for less than double the increase in the compression level.

The significance of a maximum difference value of 0.05 mm depends on the specific application. Section 3.3 analyzes how the compression level affected the rail geometry measurement, specifically addressing the acceptable maximum difference values between the compressed and raw scan points.

**Figure 13:**
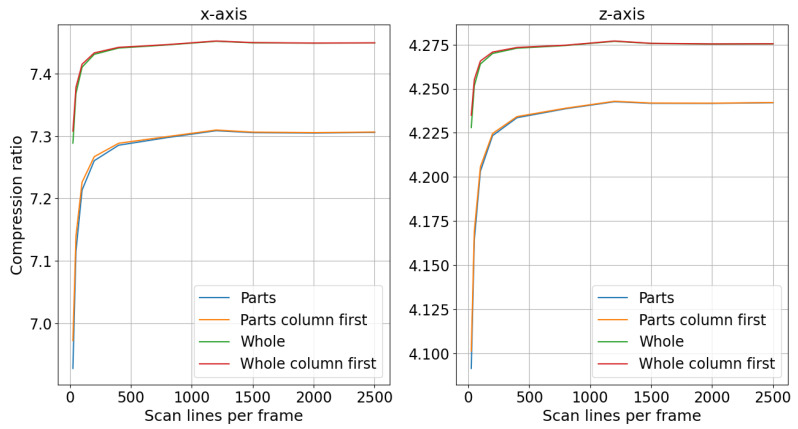
Rail geometry scan compression level change with frame size and concatenation of sensor measurements.

Similar to rail track inspection scans, the compression of the rail geometry scans was evaluated for various frame sizes by comparing the concatenation of frames to saved scans from each sensor independently. In Figure 13, the compression level of the rail geometry scans is plotted against the frame size. As with the rail track inspection scans, the frame size had little effect on the compression level when the frames contained a sufficient number of scan lines. For smaller frames, the x-axis compression ratio increased from 6.9 to 7.3 as the number of lines per frame rose from 25 to 1200 when each sensor’s frame was saved independently. A smaller increase was observed for the z-axis compression ratio, which rose from 4.10 to 4.24 over the same range of lines per frame. Likewise, concatenating the scans before compression yielded better compression results, similar to the findings for the rail track inspection scans. The compression ratios reached 7.45 for the x-axis and 4.27 for the z-axis with 1200 scan lines per frame when the frames were concatenated.

### 3.3. Scan Compression Effect on Rail Geometry Computation

This section delves into the analysis of how the scan compression impacted the repeatability and reproducibility of the rail geometry measurements. By the requirements outlined in the rail geometry norm EN 13848-1 [34], each rail geometry parameter must be measured with repeatability and reproducibility of measurements below the specified limits in EN 13848-2 [38]. Repeatability refers to the 95th percentile of the difference between two measurements of rail geometry parameters on the same track under identical measuring conditions. Reproducibility, on the other hand, is defined as the 95th percentile of the difference between two measurements of rail geometry parameters on the same track but under different measurement conditions, as outlined in EN 13848-2 [38]. A minimum of sixteen measurements of the same track section are required to conduct a comprehensive EN 13848-1 [34] repeatability and reproducibility compliance test.

**Figure 14:**
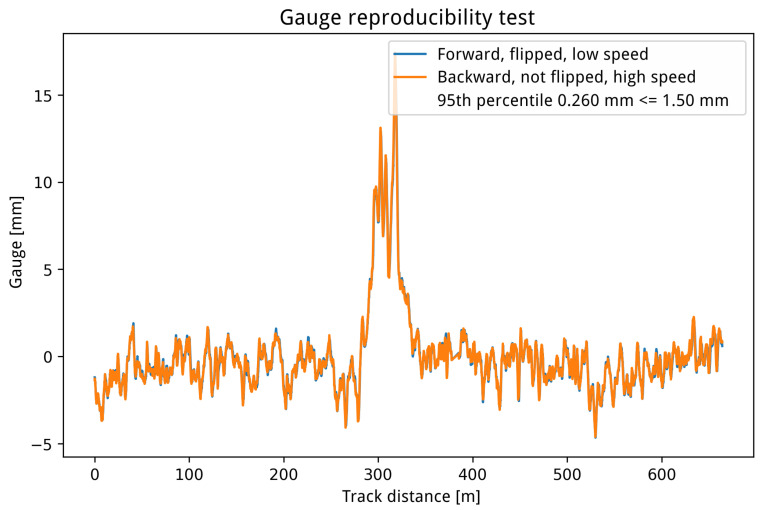
One of the rail gauge measurement reproducibility tests on a 700-meter track section, with a 95th percentile computation of two signal samples’ absolute differences.

To assess the impact of compression on the rail geometry measurement, the reproducibility of gauge measurement for rail scans compressed at different numerical precision scaler values was computed. One of the reproducibility tests, the results of which are depicted in Figure 14, was used. This test compared the absolute differences between samples of two measurements conducted under different conditions. The first measurement was acquired at a low vehicle speed while traveling in the rising direction of the track, with the vehicle oriented in the falling direction. In contrast, the second measurement was taken at high vehicle speed in the falling direction, with the vehicle oriented in the rising direction. Figure 14 illustrates the gauge signals for both measurements, expressed as the difference from the standard track gauge of 1435 mm. The 95th percentile of the gauge signals for these two measurements was 0.26 mm.

**Figure 15:**
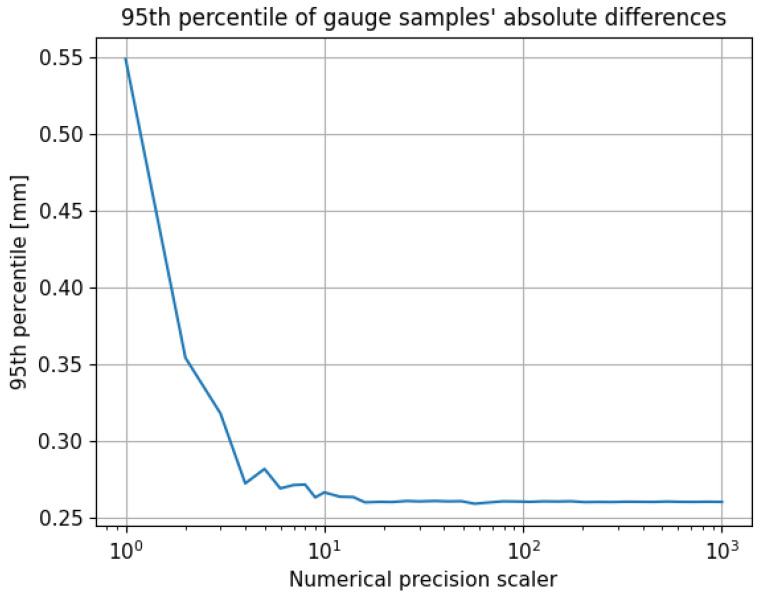
The 95th percentiles of gauge signal samples’ absolute differences as a function of the numerical precision scaler.

To evaluate the compression effect, scans from one measurement signal were compressed while the scans from another signal remained uncompressed. The 95th percentile of the absolute difference values between the gauge samples from the compressed and uncompressed scans is plotted in Figure 15. The 95th percentile increased as the numerical precision scaler values decreased, particularly growing significantly for values below 20. At a numerical precision scaler value of 20, the smallest preserved value in compression was 0.05 mm. For numerical precision scalers below four, the difference between the two signals increased rapidly. The smallest preserved fraction for a numerical precision scaler of four was 0.25. For instance, if the absolute difference before the compression between two samples was 0.26 mm, with values of 7.75 mm and 7.49 mm, compressing the sample of 7.49 mm with a numerical precision scaler of four resulted in a stored value of 7.25 mm, causing the difference to grow to 0.5 mm. As the numerical precision scaler decreased, the number of possible fractional values diminished, leading to a greater influence of rounding in the compression process compared with the actual measured sample values.

Considering that the laser scanner used had an accuracy measurement between 0.1 and 0.01 mm within this measurement range, the 95th percentile remained largely unaffected for numerical precision scaler values above 20. Although the difference between the signals increased, it did not surpass the differences already in the raw signals, which resulted in the 0.26-millimeter 95th percentile value. The EN13848 norm requires the 95th percentile for a gauge reproducibility test to be below 1.5 mm [38]. The reproducibility of the rail gauge measurement in this test remained unaffected for numerical precision scaler values not smaller than 20. Figure 12 illustrates that employing a numerical precision scaler value of 20 yielded a compression ratio of approximately 7.5.

## 4. Prospects

The compression of point cloud data measured by triangulation scanners is relatively straightforward compared with other sensors, like LiDAR. The structured nature of rail scan data lends itself well to compression techniques, with the rounding of measurement fractions notably increasing the compression ratio without a significant loss of data quality. This was qualitatively demonstrated in the rail track inspection scans and quantitatively validated in the rail geometry scans.

It is crucial to contextualize the importance of data compression within the broader framework of data acquisition and processing in rail infrastructure inspection. The raw data generated by sensors undergo processing within the data pipeline, where defects and foreign objects are detected, or geometric quantities of the rail track are measured. Detected defects in the positions and labels or computed geometric quantities expressed as floating-point numbers are the best compression possible; only information of interest is retained. However, detection algorithms are susceptible to miss predictions, and a mere label might not suffice for human operators without confirmation from the point cloud render. Similarly, variations in environmental conditions, like snow or grease, can lead to erroneous point selection for the geometry parameter computation, resulting in significant deviations from the norm. Storing compressed scans for sections where geometry parameters deviate notably from historical data or norms could prove beneficial.

Another aspect to consider is the development of detection algorithms. Whether relying on classical or machine learning techniques, data collection and storage are essential for algorithm training and validation.

Beyond space-saving advantages, data compression facilitates faster data transmission, whether wirelessly over mobile networks for sending alerts or downloading data directly from measuring devices that may be too large, even after compression for mobile network transmission. The proper selection of the numerical precision scaler in compression algorithms enables the customization of compression levels for various purposes. For instance, scans intended to alert rail operators of significant defects could be compressed with a smaller numerical precision scaler, while data reserved for training could be compressed with a larger scaler value.

## 5. Conclusions

This paper presents a straightforward triangulation sensor scan compression algorithm that leverages the widely used PNG standard for image compression. The algorithm supports various compression levels, including both lossless and lossy options. It was tested on data collected from the rail track inspection module, which utilized seven scanners, as well as scans from the rail-geometry-measuring module, which employed six scanners. To the best of the authors’ knowledge, this is the first solution presented for the compression of data from triangulation scanners and rail track inspection.

By adjusting the numerical precision scaler—a key parameter of the algorithm—a tradeoff could be achieved between the compression ratio and the resolution of the compressed measurements by effectively varying the number of retained digits in the measured sample fraction. For lossless compression, the achieved compression ratios were 3.8 for the rail track inspection scans and 5.1 for the rail geometry scans. After the qualitative analysis, the compression ratio for the rail track inspection scans was increased to 5.6 without any noticeable degradation in the scan quality. Quantitative analysis further allowed the compression ratio for the rail geometry scans to be raised to 7.5 while maintaining consistent reproducibility test results.

This algorithm is particularly advantageous for rail track inspection and rail geometry measurement devices, where extensive data collection is common. Its benefits include the efficient storage of measured data, streamlined data transmission, and the ability to send compressed point clouds containing detected defects over mobile networks to rail operators, facilitating rapid decision-making processes. Although designed primarily for rail inspection systems, this algorithm can also be applied to any scans measured by triangulation laser scanners.

## Data Availability

Data unavailable for public sharing due to privacy reasons.
